# Supplementary material for: ER O-glycosylation in synovial fibroblasts drives cartilage degradation
Source: Nat Commun. 2025 Mar 14;16:2535. doi: 10.1038/s41467-025-57401-9 (PMC11909126; doi:10.1038/s41467-025-57401-9)
Supplement: Supplementary file 1 — Supplementary Information [file 41467_2025_57401_MOESM1_ESM.pdf]

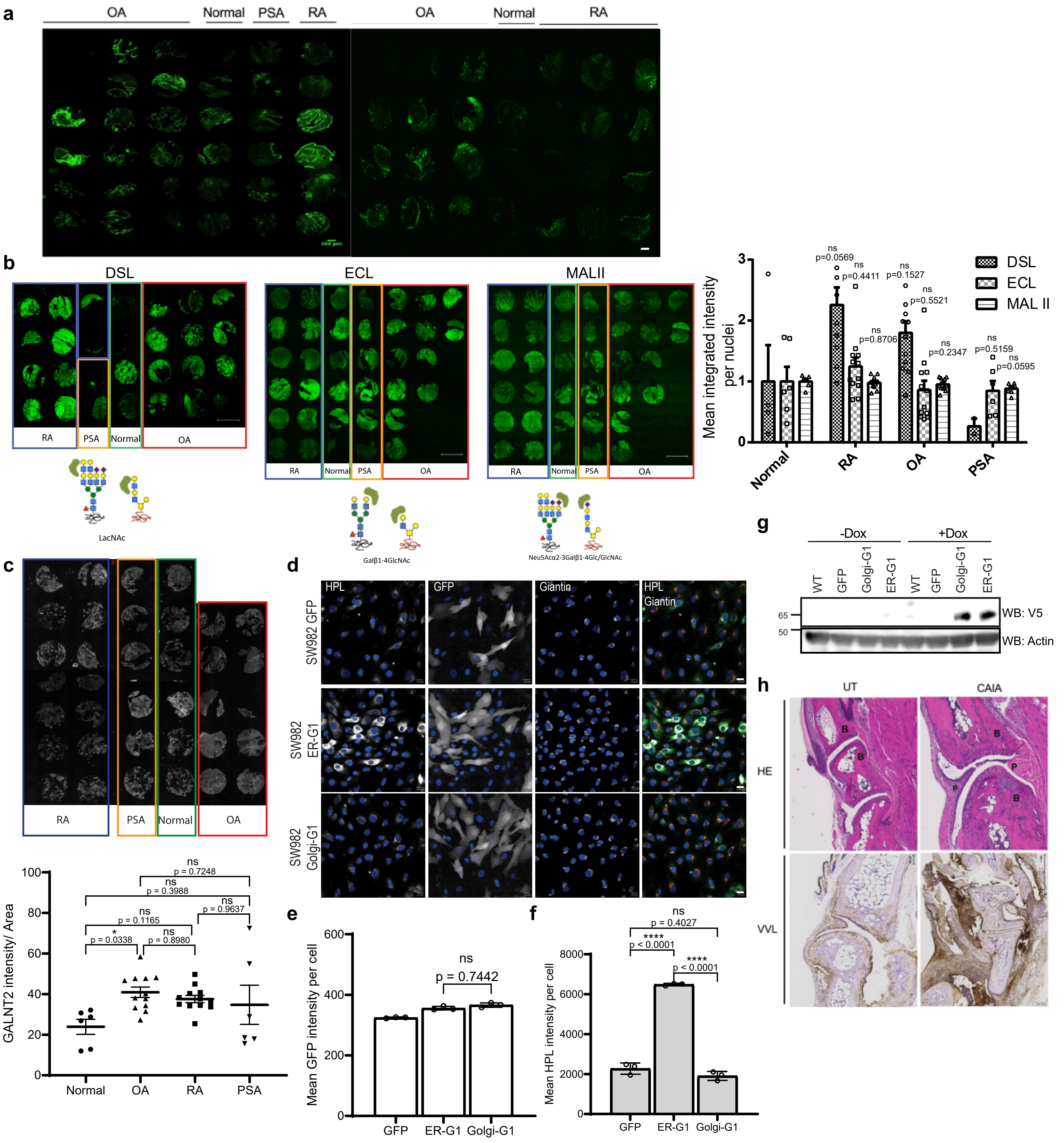

Supplementary Fig. 1: O-GalNAc (Tn) glycans tissue analysis in synovial tissues from both arthritis patients and arthritis induced animals.
(a) Representative immunofluorescence (IF) staining of O-GalNAc glycans with VVL lectin on human tissue microarray (TMA) sections from Osteoarthritis (OA), psoriasis (PSA), rheumatoid arthritis (RA) and health subjects (Normal). Magnification 10X. Scale bar, 500 µm. (b) IF staining of human tissue array with lectins DSL (Datura Stramonium Lectin), ECL (Erythrina Cristagalli Lectin) and MAL II (Maackia Amurensis Lectin). Glycan structures targeted by the lectins were shown below each panel. Quantification of the lectin staining intensities shown on the right. Data are shown as mean ± SD. The p-values presented are statistical tests comparing RA, OA or PSA with respect to normal tissues for each lectin stain. ns, not significant (Two sided Student's t test, two-tailed p-value). (c) IF staining of human tissue array with GALNT2 antibody. Quantification of the staining intensities shown below. *, p<0.05 (p=0.0338; Normal vs OA); ns, not significant (One-way ANOVA test). (d) Representative images of Tn staining using HPL in SW982 cell lines expressing control GFP, ER-localised GALNT1 (ER-G1) and wildtype GALNT1 (Golgi-G1). Scale bar, 20 µm. (e) Quantification of total GFP fluorescence intensity in control GFP, ER-G1-GFP and Golgi-G1-GFP expressing SW982 cells. ns, not significant (p=0.7442; ER-G1 vs Golgi-G1) (One-way ANOVA test). (f) Quantification of HPL intensity in GFP, ER-G1 and Golgi-G1 expressing SW982 cells. Data are shown as mean ± SD. ****, p<0.0001; ns, not significant (p=0.4027; GFP vs Golgi-G1) (One-way ANOVA test). (g) Levels of exogenously expressed GALNT1 in wildtype SW982 (WT), control GFP (GFP), wildtype GALNT1 (Golgi-G1) and ER-localised GALNT1 (ER-G1) that were uninduced (-Dox) or induced with 1µg/ml doxycycline (+Dox) over 24 hours. Exogenously expressed GALNT1 has a V5 tag. (h) HE histology (upper panel) and immunohistochemistry staining with VVL lectin (lower panel) on collagen type II antibody induced arthritis (CAIA) mice at day 7 or those left untreated (UT).

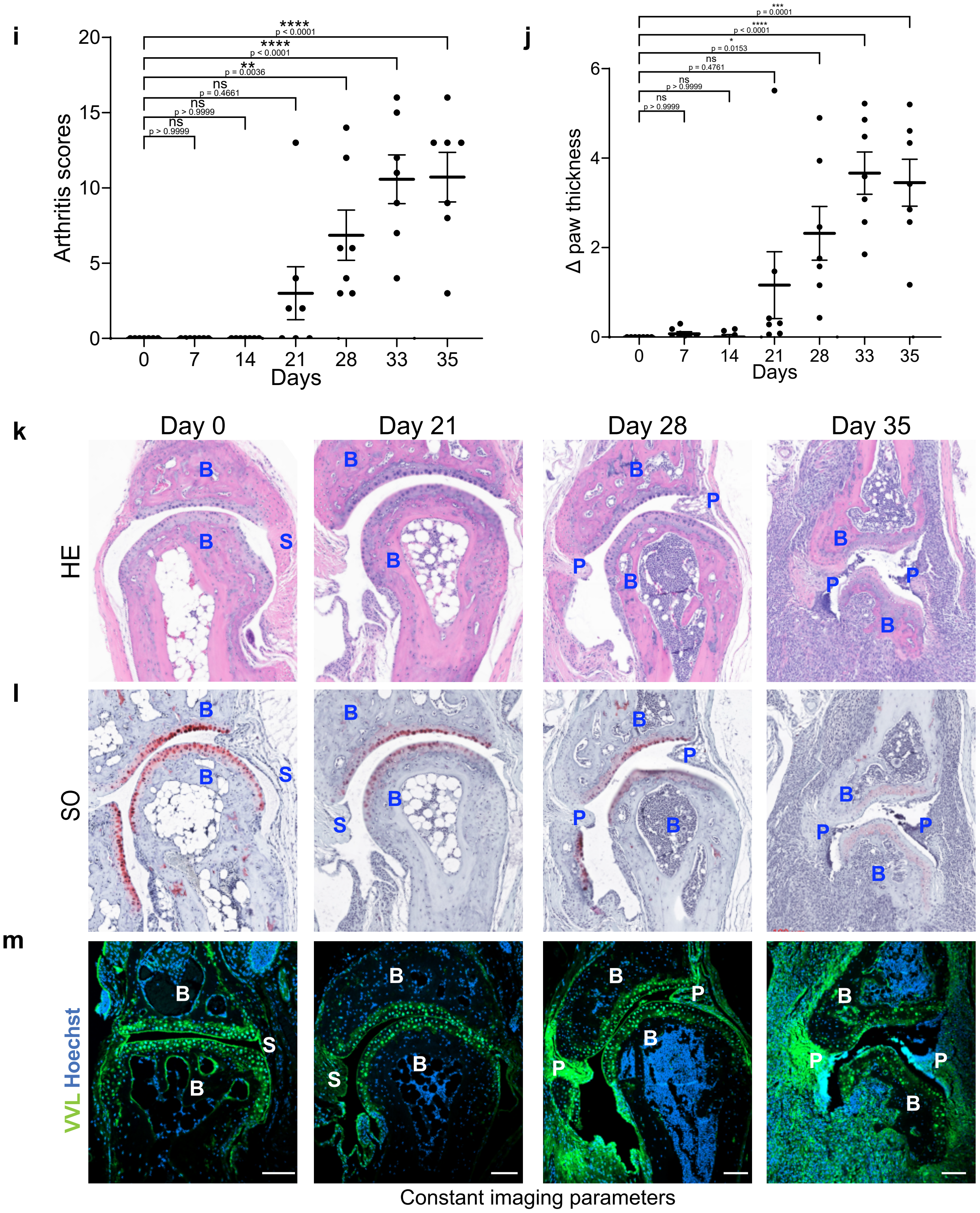

Supplementary Fig. 1: O-GalNAc (Tn) glycans tissue analysis in synovial tissues from both arthritis patients and arthritis induced animals
(i) Clinical scores of CIA mice (n=7) from day 0 to day 35. Mean ± SD of the arthritic scores of mice per time point presented. **, p<0.01 (p=0.0036; day 28); ****, p<0.0001 (day 33 and 35); ns, not significant (p>0.9999; day 7 and 14 and p=0.46661; day 21) (Two sided Student's t test, two-tailed p-value).
(j) Measurement of the change in paw thickness in CIA mice from day 0 to day 35. Mean ± SD of 7 mice per time point presented. *, p<0.05 (p=0.0153; day 28); ****, p<0.0001 (day 33 and 35); ns, not significant (p>0.9999; day 7 and 14 and p=0.4761; day 21) (Two sided Student's t test, two-tailed p-value).
(k) Hematoxylin and eosin (HE) histology images of synovial tissues sections from control (day 0) or CIA mice at day 21, 28 and 35. S: synovium; B: bone, P: pannus. (l) Safranin O (SO) histology images of synovial tissues sections from control (day 0) or CIA mice at day 21, 28 and 35. (m) VVL lectin (green) and nuclei (Hoescht) images of synovial tissues sections from control (day 0) or CIA mice at day 21, 28 and 35. Images were acquired under constant parameters. Scale bar, 100 µm. Notice strongly VVL stained pannus (P) at day 28 and 35.

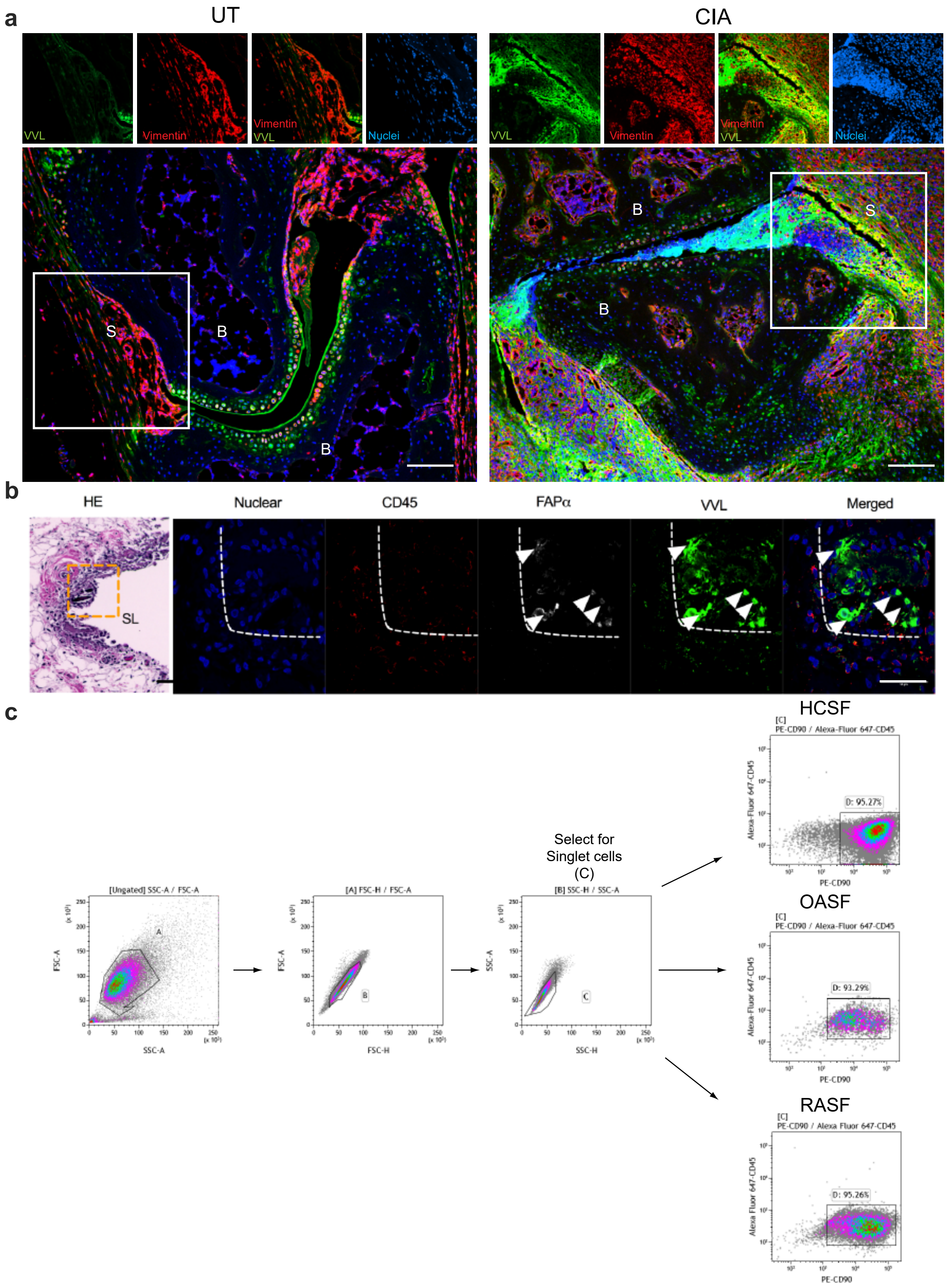

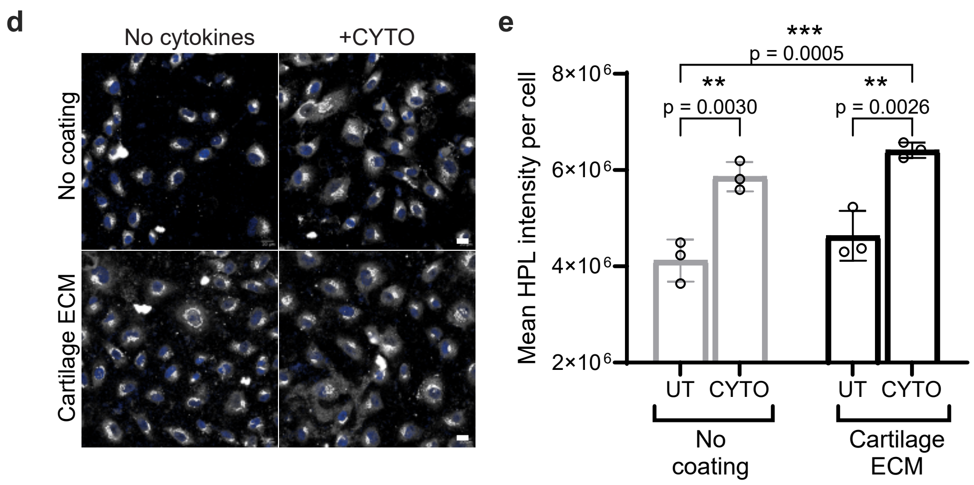

Supplementary Fig. 2: Synovial tissue analysis and primary synovial fibroblasts purification metric. (a) Representative immunofluorescence images of joints from CIA mouse on day 28 post induction. Mouse fibroblasts are stained with fibroblast maker vimentin and nuclei with hoescht staining; Scale bar, 50 µm. S: synovium; B: bone. (b) (left) (H&E) histology of synovial tissues obtained from OA patients. Scale bar, 100 µm; SL: synovial lining. (right) Representative immunofluorescence images of OA synovium stained with VVL/CD45 and FAPa. Lining synovial fibroblasts are identified by FAPa (arrowheads).Immune cells are identified by CD45. Sublining layer and lining layers boundaries are defined with dash line. Scale bar, 50 µm. (c) Gating strategy of the flow cytometry analysis of primary SF cultures from synovial tissues of healthy subjects (HCSF), OA (OASF) and RA (RASF) patients. Dot plots showing high purity (>90%) of fibroblast cells. (d) Representative images of wildtype SW982 cells stimulated with or without CYTO and cartilage ECM stained with HPL lectin in white. Scale bar: 20 µm. (e) Quantification of HPL intensity per cell in wildtype SW982 under basal conditions (UT) and upon presence of cytokines IL1β and TNFα (CYTO) plated on plastic (No coating) or plated on cartilage ECM. Data are mean ± SD of three replicates. **, $p < 0.01$ ($p=0.003$; UT vs CYTO, No coating and $p=0.0026$; UT vs CYTO, Cartilage ECM coating), ***, $p < 0.001$ (One-way ANOVA test).

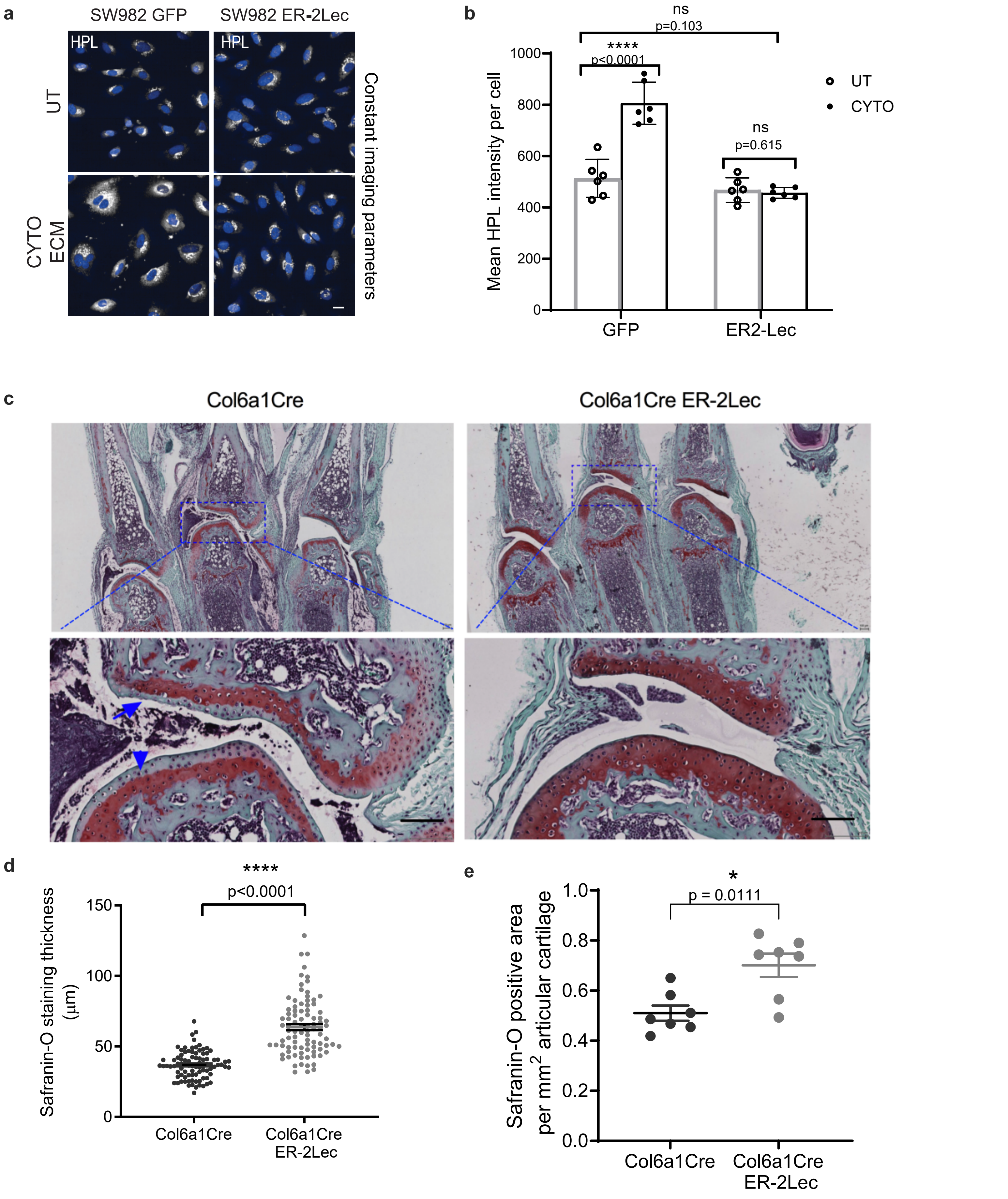

Supplementary Fig. 3: GALA activation causes damage to cartilage in CAIA mice. (a) Tn staining in SW982 cells expressing GFP or ER-2Lec. Scale bar, 20 μ m. (b) Quantification of Tn levels in (a). Data are the mean ± SEM from 3 replicate wells from each of two independent experiments. ***, $p<0.001$; ns, not significant (Two sided Student's t test, two-tailed p-value). (c) Representative images of Safranin-O (SO) staining in untreated Col6a1Cre mice or arthritis induced Col6a1Cre and Col6a1Cre ER-2Lec mice at day 7. Scale bars, 100 μm. (d & e) Quantification of SO staining thickness (d) and total positive staining area (e). Arthritis induced cartilage matrix degradation is indicated with arrowheads. Each data point represents an average of positive staining area per mm2 articular cartilage from different metacarpophalangeal joints of five animals. Data are shown as mean ± SEM. *, $p<0.05$ ($p=0.0111$); ****, $p<0.0001$ (Mann-Whitney test, two-tailed p-value).

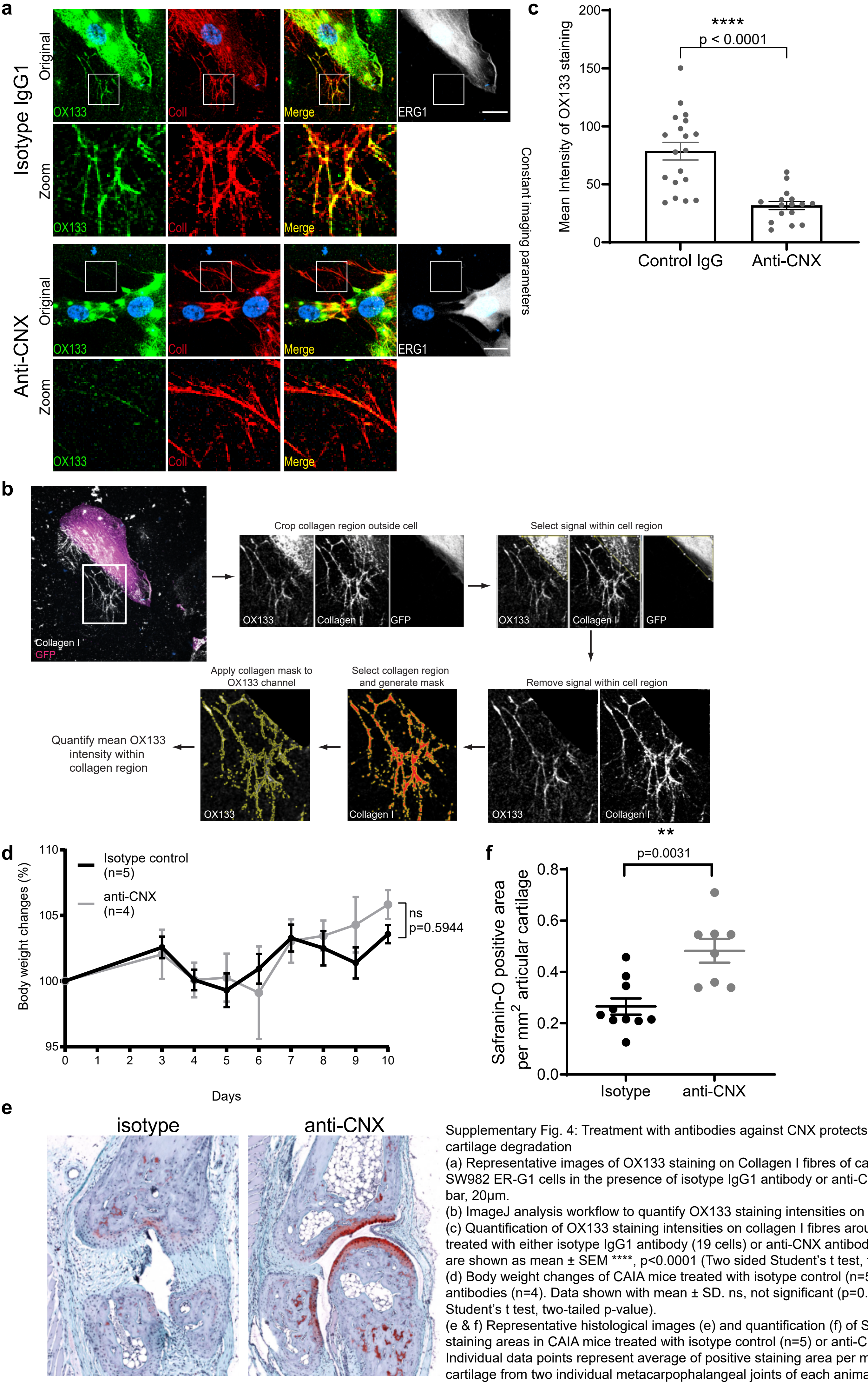

Supplementary Fig. 4: Treatment with antibodies against CNX protects CAIA mice from cartilage degradation

(a) Representative images of OX133 staining on Collagen I fibres of cartilage ECM around SW982 ER-G1 cells in the presence of isotype IgG1 antibody or anti-CNX antibody. Scale bar, 20μm.

(b) ImageJ analysis workflow to quantify OX133 staining intensities on collagen I fibres.

(c) Quantification of OX133 staining intensities on collagen I fibres around cells that were treated with either isotype IgG1 antibody (19 cells) or anti-CNX antibody (16 cells). Data are shown as mean ± SEM ****, p<0.0001 (Two sided Student's t test, two-tailed p-value).

(d) Body weight changes of CAIA mice treated with isotype control (n=5) or anti-CNX antibodies (n=4). Data shown with mean ± SD. ns, not significant (p=0.5944) (Two sided Student's t test, two-tailed p-value).

(e & f) Representative histological images (e) and quantification (f) of Safranin O (SO) staining areas in CAIA mice treated with isotype control (n=5) or anti-CNX (n=4) antibodies. Individual data points represent average of positive staining area per mm2 articular cartilage from two individual metacarpophalangeal joints of each animal. Data are shown as mean ± SEM. **, p<0.01 (p=0.0031) (Mann-Whitney test, two-tailed p-value).

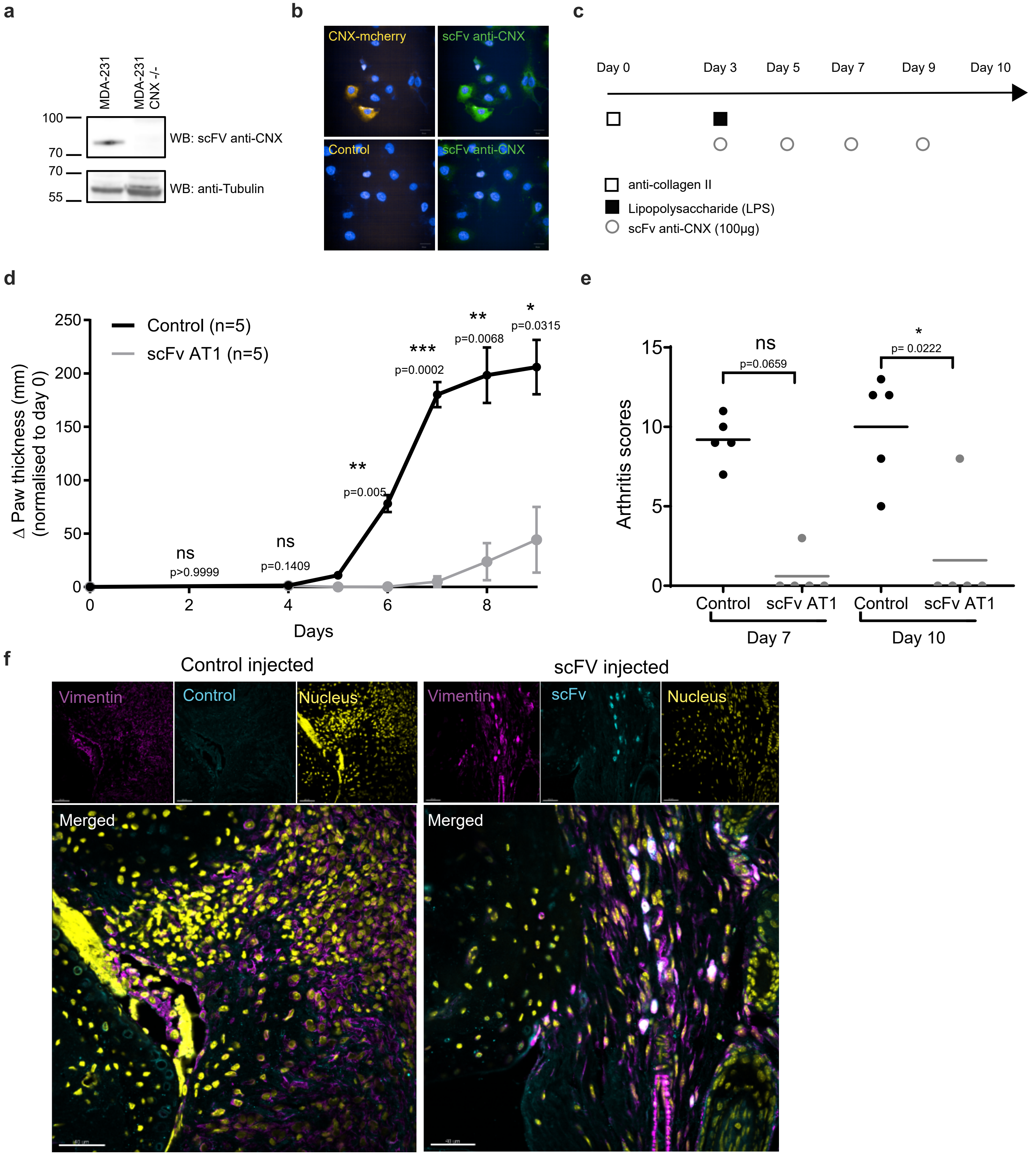

Supplementary Fig. 5: Blockage of CNX with single chain Fv (scFv) protects CAIA mice from arthritis.
(a) Western Blot analysis of CNX with identified scFv against CNX. Cell lysates from MDA-231 and MDA-231 CNX-/- probed with myc tagged scFv specific for CNX and subsequently with anti-myc Horseradish peroxidase. A loading control with tubulin is indicated. (b) Representative immunofluorescence images with scFv against CNX. Fixed and permeabilized Huh7 normal cells or Huh7 cells with stably integrated mcherry CNX (orange) subjected to incubation with myc tagged scFv and subsequently to secondary anti-Myc conjugated to PhycoErythrin (green) and Hoechst nuclear dye (blue). Representative pictures for CNX mcherry stable cells are indicated on top (left) along with scFv co-stain (right). Representative pictures for Huh7 control cells are indicated on the bottom (left) along with scFv co-stain (right). All images are from constant acquisition and display settings. Scale bar, 20µm. (c) Experimental setup of injections and timeline used for treatment of collagen antibody induced arthritis mouse model with scFv against CNX. (d) Paw thickness variation in CAIA mice treated with scFv injection or control PBS injection from day 0 to day 10. Data represent mean ± SEM, n=5 mice injected with isotype antibody or with scFv antibody. *, $p<0.05$ ($p=0.0315$; day 9); **, $p<0.01$ ($p=0.005$; day 6 and $p=0.0068$; day 8); ***$<p<0.001$ ($p=0.0002$; day 7); ns, not significant ($p>0.9999$; day 2 and $p=0.1409$; day 4) (Two-way ANOVA test).(e) Clinical scores of CAIA mice treated with scFv injection or control PBS injection from day 0 to day 10. *, $p<0.05$ ($p=0.0222$; day10); ns, not significant ($p=0.0659$; day 7) (Two-way ANOVA test). (f) Representative immunofluorescence images of synovial tissues sections from CAIA mice day 10 from control PBS or myc tagged scFv injected mice stained with anti-Myc conjugated to PhycoErythrin (green), Vimentin and DAPI. Scale bar, 40 µm.

Table 1: Antibody list

No.	Ab name	Supplier name	CAT number	Clone	Amount used
1	Anti-CNX	Abcam	ab10286	Rabbit IgG, polyclonal	1/100
2	Anti-CNX	Abcam	ab22595	Rabbit IgG, polyclonal	1/100
3	Anti-Vimentin	Abcam	ab92547	EPR3776	1/200
4	Anti-beta actin	Abcam	ab8226	mAbcam 8226	1/500
5	PECy7 conjugated anti-CD45	Biolegend	103113	30-F11	1/200
6	PE conjugated anti-Human CD90	Biolegend	328110	5E10	1/100
7	Anti-FAPα	R&D systems	MAB3715	427819	1/100
8	Anti-NEM OX133	Absolute Antibody	Ab00579-1.1	OX-133	1/100
9	Anti-GALNT2	Abcam	ab262868	Rabbit IgG, polyclonal	1/100
10	Anti-GALNT2	Novus	NBP1-83394	Rabbit IgG, polyclonal	0.25-2 ug/ml
11	Anti-TGN46	Abcam	ab16059	Rabbit IgG, polyclonal	1/200
12	anti-TGN46	AbD Serotec	AHP500G	Sheep IgG, polyclonal	1/200
13	Anti-Giantin	Abcam	ab37266	9B6	1/200
14	Anti-PDIA3 (Anti-ERp57)	Abcam	ab13506	MaP.Erp57	1/200
15	Anti-PDIA4 (Anti-ERp72)	Abcam	ab155800	Rabbit IgG, polyclonal	1/200
16	Anti-PDIA4 (Anti-ERp72)	Abcam	ab82587	Rabbit IgG, polyclonal	1/200
17	Anti-Collagen I	Southern Biotech	1310-01	Goat IgG, polyclonal	1/500
18	Anti-Collagen III	Abcam	ab7778	Rabbit IgG, polyclonal	1/100
19	Anti-Fibronectin	Abcam	ab2413	Rabbit IgG, polyclonal	1/100
20	anti-Calreticulin	Abcam	ab22683	Mouse IgG, monoclonal	1ug/ml
21	Rabbit polyclonal isotype control	Abcam	ab37415	Rabbit IgG, polyclonal	1/100
22	Anti-rabbit IgG-HRP	GE Healthcare Life Sciences	NA934	Goat IgG	1/400
23	Anti-mouse IgG-HRP	GE Healthcare Life Sciences	NA931	Sheep IgG	1/400
