# Supplementary material for: ER O-glycosylation in synovial fibroblasts drives cartilage degradation
Source: Nat Commun. 2025 Mar 14;16:2535. doi: 10.1038/s41467-025-57401-9 (PMC11909126; doi:10.1038/s41467-025-57401-9)
Supplement: Supplementary file 2 — Reporting Summary [file 41467_2025_57401_MOESM2_ESM.pdf]

nature portfolio

Corresponding author(s): Frederic Bard

Last updated by author(s): 4th February 2025

Reporting Summary

Nature Portfolio wishes to improve the reproducibility of the work that we publish. This form provides structure for consistency and transparency in reporting. For further information on Nature Portfolio policies, see our Editorial Policies and the Editorial Policy Checklist.

Statistics

For all statistical analyses, confirm that the following items are present in the figure legend, table legend, main text, or Methods section.

n/a	Confirmed	
☐	☒	The exact sample size (*n*) for each experimental group/condition, given as a discrete number and unit of measurement
☐	☒	A statement on whether measurements were taken from distinct samples or whether the same sample was measured repeatedly
☐	☒	The statistical test(s) used AND whether they are one- or two-sided<br>*Only common tests should be described solely by name; describe more complex techniques in the Methods section.*
☐	☒	A description of all covariates tested
☐	☒	A description of any assumptions or corrections, such as tests of normality and adjustment for multiple comparisons
☐	☒	A full description of the statistical parameters including central tendency (e.g. means) or other basic estimates (e.g. regression coefficient) AND variation (e.g. standard deviation) or associated estimates of uncertainty (e.g. confidence intervals)
☐	☒	For null hypothesis testing, the test statistic (e.g. *F*, *t*, *r*) with confidence intervals, effect sizes, degrees of freedom and *P* value noted<br>*Give P values as exact values whenever suitable.*
☒	☐	For Bayesian analysis, information on the choice of priors and Markov chain Monte Carlo settings
☒	☐	For hierarchical and complex designs, identification of the appropriate level for tests and full reporting of outcomes
☒	☐	Estimates of effect sizes (e.g. Cohen's *d*, Pearson's *r*), indicating how they were calculated

Our web collection on statistics for biologists contains articles on many of the points above.

Software and code

Policy information about availability of computer code

Data collection	No coding software was used to collect the data.
Data analysis	Confocal images were analyzed using Image J software, version 2.2.2-rc-69/1.52i. Automated microscope images were analyzed with Columbus software, version 2.9.1.699. Data were analyzed using GraphPad Prism software, version 10.0.0 (153).

For manuscripts utilizing custom algorithms or software that are central to the research but not yet described in published literature, software must be made available to editors and reviewers. We strongly encourage code deposition in a community repository (e.g. GitHub). See the Nature Portfolio guidelines for submitting code & software for further information.

Data

Policy information about availability of data

All manuscripts must include a data availability statement. This statement should provide the following information, where applicable:

- Accession codes, unique identifiers, or web links for publicly available datasets
- A description of any restrictions on data availability
- For clinical datasets or third party data, please ensure that the statement adheres to our policy

All data supporting the findings described in this manuscript are available in the article and in the Supplementary Information and from the corresponding author upon request. Source data are provided with this paper.

Research involving human participants, their data, or biological material

Policy information about studies with human participants or human data. See also policy information about sex, gender (identity/presentation), and sexual orientation and race, ethnicity and racism.

Reporting on sex and gender	Fresh synovial tissues were obtained based on the diagnostic criteria of RA and OA at TTSH. There were two RA patients (male aged above 50 years and female aged above 65) and three OA patients (two males and a female aged above 65). For the TMA samples, the gender of the patients and healthy individuals can found on Provitro AG website. The tissues were from an equal mix of both gender. These information was reported in materials and methods section.
Reporting on race, ethnicity, or other socially relevant groupings	For the TTSH samples, all the patients were Chinese except one OA patient who was Malay. For the TMA samples, the ethnicity of the patients were also not publicly available in the datasheet and website. These information was reported in materials and methods section.
Population characteristics	Synovial tissues were obtained from 5 patients that met the diagnostic criteria of RA and OA at TTSH and the representative data was shown on the figures. The TMA samples comprises of tissues from 21 OA patients, 18 RA patients, 6 PSA patients and 7 healthy subjects.
Recruitment	Synovial tissue specimens were obtained from patients with rheumatoid arthritis (RA) and osteoarthritis (OA) undergoing joint replacement surgery at the TTSH. The collection of samples will only be done as part of patients' standard clinical care. Patients gave written consent and met the diagnostic criteria for RA and OA. There was no self-selection bias or other biases that may be present.
Ethics oversight	The procedures were approved by the Ethics Committee of The National Healthcare Group domain specific review board under protocol no. 2018/00980.

Note that full information on the approval of the study protocol must also be provided in the manuscript.

Field-specific reporting

Please select the one below that is the best fit for your research. If you are not sure, read the appropriate sections before making your selection.

☒ Life sciences ☐ Behavioural & social sciences ☐ Ecological, evolutionary & environmental sciences

For a reference copy of the document with all sections, see nature.com/documents/nr-reporting-summary-flat.pdf

Life sciences study design

All studies must disclose on these points even when the disclosure is negative.

Sample size	The exact sample size of each experiment is described in the text and relevant figure legends.
Data exclusions	Cells of low cell viability were excluded from flow cytometric analysis.
Replication	All experiments were performed with at least two biological replicates.
Randomization	Randomization was not performed and not relevant to this study.
Blinding	Scoring of arthritis symptoms in experiments involving the CAIA and CIA mouse models was performed by a researcher who is blinded on the treatment given.

Reporting for specific materials, systems and methods

We require information from authors about some types of materials, experimental systems and methods used in many studies. Here, indicate whether each material, system or method listed is relevant to your study. If you are not sure if a list item applies to your research, read the appropriate section before selecting a response.

Materials & experimental systems

n/a	Involved in the study	
☐	☒	Antibodies
☐	☒	Eukaryotic cell lines
☒	☐	Palaeontology and archaeology
☐	☒	Animals and other organisms
☒	☐	Clinical data
☒	☐	Dual use research of concern
☒	☐	Plants

Methods

n/a	Involved in the study	
☒	☐	ChIP-seq
☐	☒	Flow cytometry
☒	☐	MRI-based neuroimaging

Antibodies

Antibodies used: Please fnd the list of antibodies, their source and dilution used in supplementary data 1

Validation: Most antibodies used in this study are commercially available and validated by their respective manufacturers. We have validated specificity of our in-house scFV against CNX and it was presented in Supplementary figure 5.

Eukaryotic cell lines

Policy information about cell lines and Sex and Gender in Research

Cell line source(s): SW982 (ATCC® HTB-93) was purchased directly from ATCC.

Authentication: Cell from commercial source was distributed with certificates of authentication using STR profiling.

Mycoplasma contamination: Regular mycoplasma testing was done for SW982 and was tested negative for mycoplasma contamination.

Commonly misidentified lines (See ICLAC register): No commonly misidentified cell line.

Animals and other research organisms

Policy information about studies involving animals; ARRIVE guidelines recommended for reporting animal research, and Sex and Gender in Research

Laboratory animals: Col6a1Cre mice expressing collagen type VI promoter driven on the C57BL/6J background were provided by G. Bressan (University of Milano, Milano, Italy). DBA/1J mice were obtained from the Jackson laboratory (USA) and the colony was expanded for experiments. All animals were bred and maintained under specific pathogen-free conditions in micro-isolator cages with access to food and water at Biological Resource Centre (ASTAR, Singapore).

Wild animals: No wild animals were involved in this study.

Reporting on sex: Only males mice were used in this study.

Field-collected samples: No field collection of samples were in this study.

Ethics oversight: Experiments were performed using age- and sex-matched animals and complied with guidelines approved by the Animal Ethics Committees at Biological Research Centre (ASTAR, Singapore) under the protocol IACUC no. 201548.

Note that full information on the approval of the study protocol must also be provided in the manuscript.

Plants

Seed stocks: Not applicable.

Novel plant genotypes: Not applicable.

Authentication: Not applicable.

Flow Cytometry

Plots

Confirm that:

☒ The axis labels state the marker and fluorochrome used (e.g. CD4-FITC).

☒ The axis scales are clearly visible. Include numbers along axes only for bottom left plot of group (a 'group' is an analysis of identical markers).

☒ All plots are contour plots with outliers or pseudocolor plots.

☒ A numerical value for number of cells or percentage (with statistics) is provided.

Methodology

Sample preparation	Joints were cut into small pieces and incubated in digestion buffer (1 mg/ml collagenase IV, and 1 mg/ml of DNase I in HBSS) for 60 min at 37C. Cells released during the digestion were filtered through 70 µm cell strainers, erythrocytes were lysed using a red blood lysis buffer. Cells were stained with live/dead Aqua viability dyes and stained with fluorochrome-conjugated antibodies and lectins.
Instrument	BD LSRII flow cytometer (BD Biosciences)
Software	Kaluza software (Beckman Coulter)
Cell population abundance	Primary cultures extracted from human synovial tissues showed >90% of the population was synovial fibroblasts.
Gating strategy	The gating strategy is shown in Supplementary figure 2.

☒ Tick this box to confirm that a figure exemplifying the gating strategy is provided in the Supplementary Information.
